# Metasurface-enhanced biomedical spectroscopy

**DOI:** 10.1515/nanoph-2024-0589

**Published:** 2025-01-20

**Authors:** Qiang Li, Shiwang Yu, Zhancheng Li, Wenwei Liu, Hua Cheng, Shuqi Chen

**Affiliations:** The Key Laboratory of Weak Light Nonlinear Photonics, Ministry of Education, School of Physics and TEDA Institute of Applied Physics, 12538 Nankai University, Tianjin 300071, China; School of Materials Science and Engineering, Smart Sensing Interdisciplinary Science Center, 12538 Nankai University, Tianjin 300350, China; The Collaborative Innovation Center of Extreme Optics, Shanxi University, Taiyuan, Shanxi 030006, China

**Keywords:** metasurface, biomedical spectroscopy, detection sensitivity, infrared spectroscopy, Raman spectroscopy

## Abstract

Enhancing the sensitivity of biomedical spectroscopy is crucial for advancing medical research and diagnostics. Metasurfaces have emerged as powerful platforms for enhancing the sensitivity of various biomedical spectral detection technologies. This capability arises from their unparalleled ability to improve interactions between light and matter through the localization and enhancement of light fields. In this article, we review representative approaches and recent advances in metasurface-enhanced biomedical spectroscopy. We provide a comprehensive discussion of various biomedical spectral detection technologies enhanced by metasurfaces, including infrared spectroscopy, Raman spectroscopy, fluorescence spectroscopy, and other spectral modalities. We demonstrate the advantages of metasurfaces in improving detection sensitivity, reducing detection limits, and achieving rapid biomolecule detection while discussing the challenges associated with the design, preparation, and stability of metasurfaces in biomedical detection procedures. Finally, we explore future development trends of metasurfaces for enhancing biological detection sensitivity and emphasize their wide-ranging applications.

## 1 Introduction

Spectroscopic techniques encompass a variety of scientific methods used to analyze the interaction between light and matter, focusing on how different substances absorb, emit, or scatter light, which is highly significant for biological sensing and detection applications [1], [2], [3], [4]. Importantly, these techniques are noninvasive or minimally invasive, allowing for analysis without compromising the structure of biological samples, which is critical for valuable or live specimens [5], [6], [7]. Spectroscopic methods, such as infrared, fluorescence, and Raman spectroscopy, enable precise quantification, composition analysis, and structural characterization of biological molecules with high sensitivity and accuracy [8], [9], [10], [11]. This precision makes them particularly valuable for biomarker detection, disease diagnosis, and related applications [12], [13], [14]. Furthermore, spectroscopic methods can rapidly process large sample volumes, significantly enhancing detection efficiency, which is essential for early disease screening and timely treatment [15], [16], [17]. Consequently, spectroscopic techniques have broad applications in biomedical research, offering diverse approaches to detecting biological molecules such as proteins, nucleic acids, and extracellular vesicles [18], [19], [20]. However, as research and clinical detection progress, some disease-related biomarkers are present at trace levels below the detection limits of conventional spectroscopic strategies. Additionally, environmental impurities can compromise the accuracy of spectral detection [21], [22]. To address these limitations, the development of new signal amplification strategies is essential to broaden the scope and enhance the efficacy of spectroscopic detection [23], [24], [25].

Metasurfaces are planar arrays composed of artificial microstructures at the subwavelength scale, supporting a wide range of local and nonlocal resonant modes, including localized surface plasmon resonance (LSPR), surface lattice resonance (SLR), guided-mode resonance (GMR), quasi-bound states in the continuum (q-BICs), and Mie resonances [26], [27], [28], [29], [30]. These resonant modes, along with their couplings, enable the localization and enhancement of light fields, offering an efficient means to enhance light–matter interactions. Notably, the resonant modes of metasurfaces and their coupling can be easily modulated by adjusting structural symmetry and parameters, allowing customizable near-field distributions and enhancements at desired wavelengths. In biomedical spectroscopy, various biological analytes possess different characteristic sizes and have unique vibrational spectra in specific wavelength bands. The diverse resonant modes supported by the metasurfaces have different evanescent field decay lengths and customizable resonant wavelengths, which just meet the spectral enhancement requirements of specific biomarkers [31], [32], [33], [34], [35], [36]. Consequently, metasurfaces have emerged as powerful platforms for enhancing biomedical sensing and spectroscopy with improved detection sensitivity, lowered detection limits, and faster detection efficiency. In addition to their capability of enhancing light–matter interactions through the confinement of light waves in the near field, metasurfaces can manipulate the amplitude, phase, and polarization of optical fields in the far field, facilitating optical multiplexing and multifunctional integration [37], [38]. The incorporation of metasurfaces into biomedical spectroscopy as enhanced substrates not only offers significant advantages for biomedical spectroscopy with improved performance but also creates opportunities for the development of highly integrated, label-free nanophotonic biosensor devices with added functionalities [39], [40], [41].

**Figure 1:**
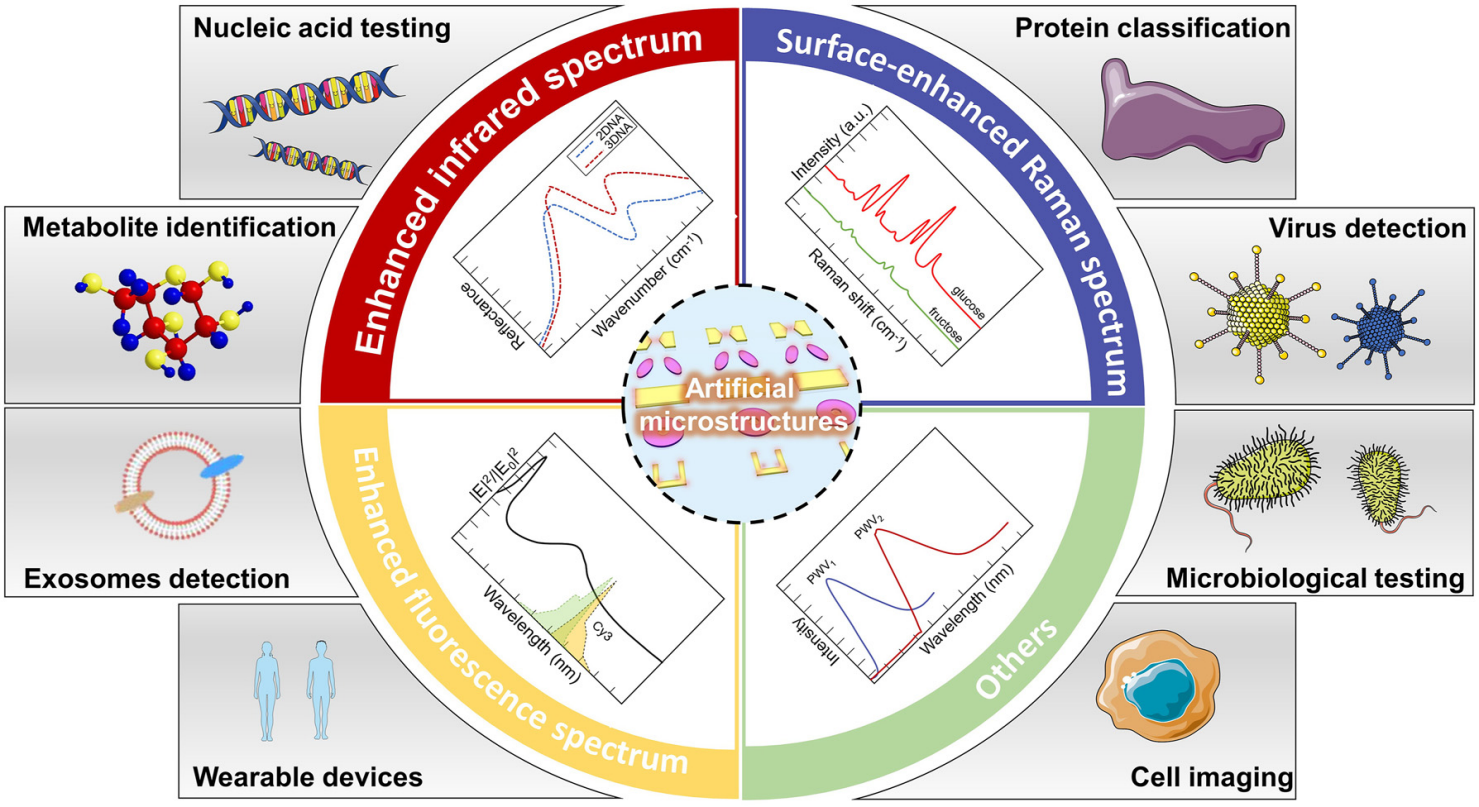
Overview of metasurface-enhanced biomedical spectroscopy. Enhanced infrared spectrum: reproduced with permission from Aditya et al., Nano Lett. 24, 11607–11614 (2024) [42]. Copyright 2024 American Chemical Society. Surface-enhanced Raman spectrum: adapted from Long et al., Nanoscale 12, 10809–10815 (2020) [43]. Copyright 2020 Royal Society of Chemistry. Enhanced fluorescence spectrum: Reproduced with permission from Radwanu et al., ACS NANO, 13, 13775–13783 (2019) [44]. Copyright 2019 American Chemical Society. Others: adapted from Hadi Shafiee et al., Sci. Rep. 4, 4116 (2024) [45]. Licensed under a Creative Commons Attribution 3.0 International License. Exosomes detection: reproduced with permission from Zhang et al., J Extracell. Vesicles, 12, e12300 (2023) [46]. Licensed under a Creative Commons Attribution 4.0 International License. Other parts were drawn by the authors.

This review focuses on metasurface-enhanced biomedical spectroscopy, offering a comprehensive discussion of various biomedical spectral detection technologies, as illustrated in Figure 1. The objective of this review is to highlight the advantages of metasurfaces in enhancing detection sensitivity, lowering detection limits, and enabling rapid biomolecule detection, while also addressing the challenges faced by metasurface-based biomedical detection methods and outlining future development trends. The review is organized as follows: In Section 2, we demonstrate the significant enhancement of infrared spectroscopy sensitivity for detecting small molecules, proteins, and lipids achieved through metasurfaces and emphasize their potential to mitigate the effects of water environments during live cell detection. In Section 3, we explore the use of metasurfaces as highly efficient substrates for Raman spectroscopy in various biomedical detection processes and their integration into wearable devices for real-time metabolite monitoring. Section 4 discusses the capacity of metasurface structures to not only improve the detection limits of fluorescence spectroscopy but also integrate seamlessly with existing fluorescence imaging devices, thereby expanding their functional capabilities. In Section 5, we outline the applications of metasurfaces in colorimetric, surface plasmon resonance (SPR), terahertz, and chiral biosensors, showcasing their significant value in biological detection. Finally, we present future trends in the advancement of metasurfaces for improving sensitivity in biological detection, highlighting their extensive potential in biomedical research and disease diagnosis.

## 2 Metasurface-enhanced infrared spectroscopy

Infrared spectroscopy, a highly effective label-free detection technique, provides valuable insights into the composition and behavior of biochemical systems by analyzing their distinct vibrational fingerprints. This nondestructive method has been widely utilized across various fields, including biology, chemistry, and medical research. However, the dipole moment associated with molecular vibrations is several orders of magnitude smaller than the infrared wavelength, resulting in very weak vibrational absorption. Consequently, a large number of biomolecules are required to achieve detectable absorption of incident infrared light. Since molecular absorption is proportional to the field strength experienced by biomolecules, an effective solution to this limitation is to leverage the enhanced optical near-field. Recent advancements in metasurfaces significantly facilitate the infrared detection of trace biomolecules due to their flexible capabilities for near-field localization and enhancement of optical waves [47], [48], [49], [50], [51].

**Figure 2:**
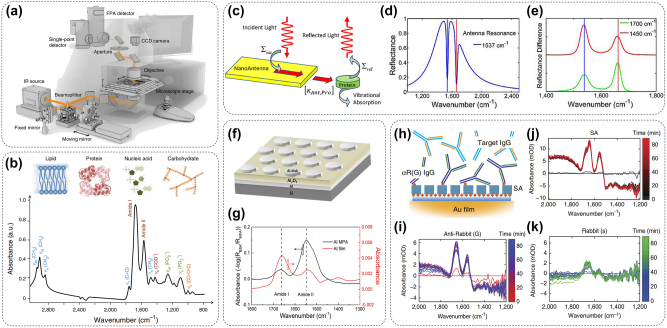
Metasurface for IR imaging in biomolecules. (a) Schematic of IR-imaging spectrometer [52]; (b) a representative IR spectrum displayed peak assignments for biomolecular vibrations within the range of 3,000 to 800 cm^−1^, categorizes these peaks according to stretching vibrations (*ν*), bending vibrations (*δ*), symmetric vibrations (*s*), and asymmetric vibrations (as) [53]. (c) The nonequilibrium Green’s function formalism describes the incident light-driven antenna and its coupling with the protein load. (d) The model replicates lines based on antenna resonance (offset) with the amide-II band (1,537 cm^−1^). (e) Difference spectrum of a resonantly tuned antenna [54]. (f) Diagram illustrating the Al metamaterial perfect absorbers (MPA) in a typical MIM configuration and (g) the absorption spectra comparison of BSA adsorbed onto Al MPAs (depicted in black) versus Al planar film (depicted in red) [55]. (h) Scheme illustrating the measurement of protein-binding interactions. A series of spectrograms were used to show (i) SA coating and enrichment efficiency for (j, k) two different IgG [56].

Infrared spectra are typically measured using microscopic inspection methods, which can be achieved by raster-scanning a spot illuminated on the sample or by employing wide-field illumination in combination with focal plane array (FPA) or linear array detectors, as illustrated in Figure 2a [52]. For biological detection, there are three primary infrared spectral sampling modes: transmission, transflection, and attenuated total reflection (ATR). As shown in Figure 2b, the most important spectral regions for infrared spectroscopy measurements are usually the fingerprint region (600–1,450 cm^−1^) and the amide I and amide II (amide I/II) region (1,500–1,700 cm^−1^). The higher wave-number (2,550–3,500 cm^−1^) region is associated with stretching vibrations such as N–H, C–H, S–H, and O–H, while the lower wave-number region generally corresponds to chemical bond bending and carbon skeleton fingerprint vibrations [53].

### 2.1 Protein detection

The correct folding of protein secondary structures and controllable conformational changes are essential for protein function [57]. Surface-enhanced infrared absorption (SEIRA) using resonant metal nanoantennas provides up to a 1,000-fold near-field intensity enhancement of amide bands, enabling protein identification and structural analysis by observing changes in the amide I (primarily C–O stretching) and amide II (a combination of C–N stretching and N–H rocking) vibrations of amino acid residues at 1,650 and 1,450 cm^−1^ [58], [59], [60]. As shown in Figure 2c, Adato et al. optimized resonant structures to match the molecular vibrational modes by tailoring the geometry of individual nanoantennas [54]. The intensity of absorption resonances was governed by the relative strength of incoming infrared radiation interacting with the protein’s vibration bands. These tailored nanoantennas are then arranged to achieve in-phase dipolar coupling (1,537 cm^−1^), resulting in collective excitation of the ensemble with a significantly enhanced near-field, yielding a signal enhancement factor of 10^4^–10^5^ (shown in Figure 2d). As shown in Figure 2e, adjusting the resonance frequency of the antenna away from its optimal point allows for the modulation of the relative absorption power observed in these vibration bands. While metasurface-empowered signal amplification enhances the intensity of the infrared absorption peak for protein detection, it also amplifies signals from other impurities. Given that the concentration of impurities in real biological samples is often much higher than that of target biomolecules, enhancing the IR signal of specific proteins to improve the signal-to-noise ratio of IR detection has emerged as a new direction for metasurface design [61]. The resonant wavelengths of metasurfaces can be easily adjusted by altering their structural parameters, enabling precise alignment with the molecular vibrations of target proteins for optimal signal enhancement. For instance, the resonant wavelengths of an aluminum (Al) nanodisk (Figure 2f) can be precisely tuned by adjusting its diameter. When these resonant wavelengths align with the infrared absorption band of target proteins, they produce a spectral lineshape with a Lorentzian profile, as shown in Figure 2g. Studies confirm that the relative strength of this absorption resonance is influenced by the coupling strength between the incident infrared radiation and the protein vibrational band [55], [62]. Modulating the structural asymmetry of the nanostructures is another effective method for tuning the resonant wavelength to align with the infrared absorption peak of specific proteins. Moreover, the gold surface is very easy to chemically modify and facilitates to adsorb of various biological detection markers such as streptavidin, protein (A/G), immunoglobulin (IgG), and specially designed peptides on the metasurface through some commercial linkers, which greatly expands the application scope of protein infrared detection (Figure 2h) [59], [63], [64], [65], [66]. Subsequently, several immunoassays based on SEIRA technology have been developed to monitor the different binding abilities between proteins and nanoparticles in real-time with high sensitivity, as shown in Figure 2i–k [56], [67], [68].

### 2.2 Lipid detection

**Figure 3:**
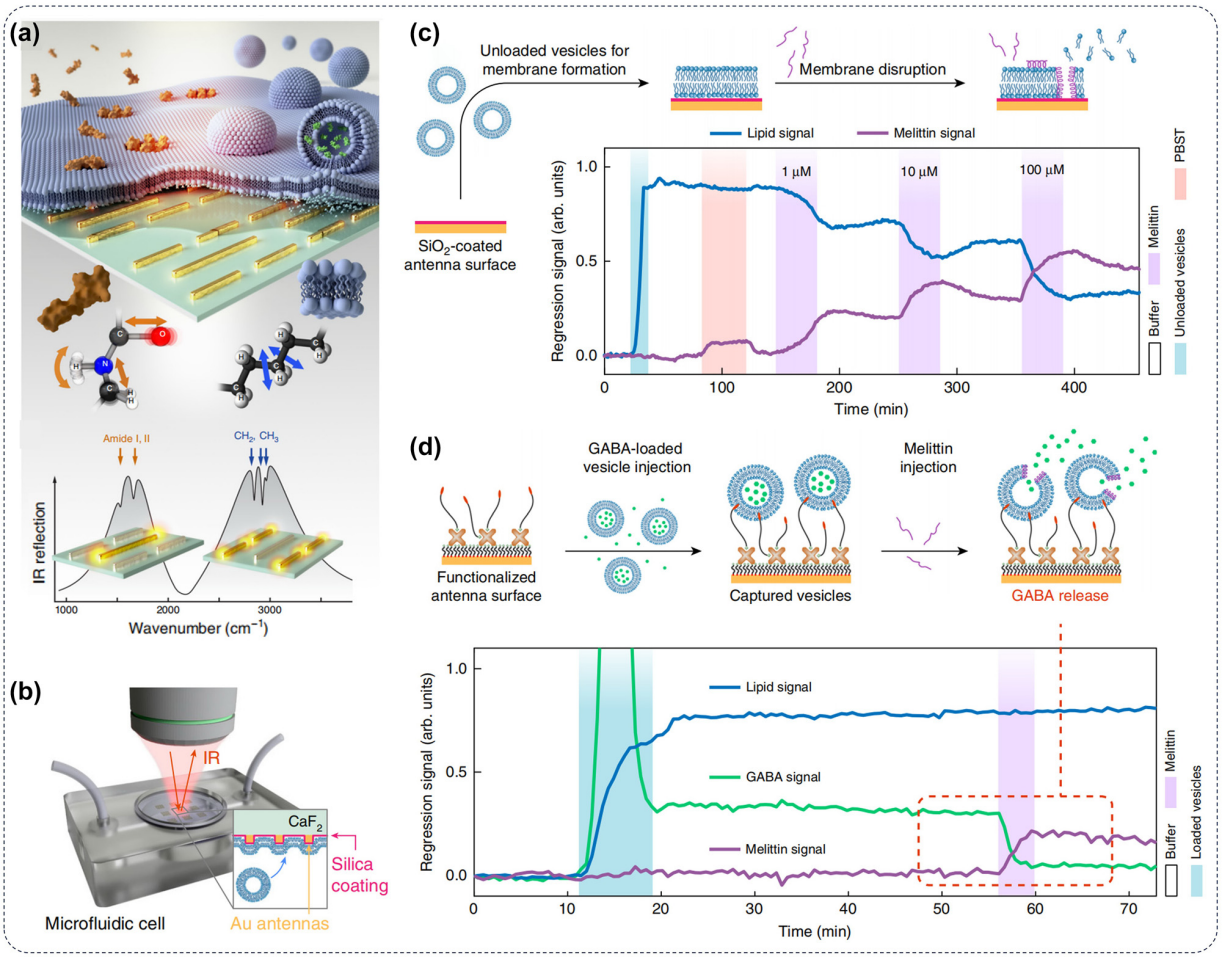
Metasurface for IR imaging of lipid structure. (a) Antenna resonance positions can serve as a label-free nanophotonic biosensor for differentiating absorption changes induced by lipids and proteins in biological samples; (b) schematic representation of phospholipid bilayer detection by IR spectroscopy; (c) the interaction of melittin with a supported lipid bilayer and the subsequent disruption of the membrane caused by increasing concentrations of melittin (1, 10, and 100 µM) are observed via IR spectroscopy; (d) an illustration outlines the experiment for vesicle cargo release, which observed by metasurface, along with time-resolved linear regression signals depicting three key biological components: lipids, GABA, and melittin [69].

Lipid is an important component of the cell membrane phospholipid bilayer, which plays an important role in phagocytosis, intracellular transport, signal transduction, and other processes [69], [70]. The alkyl structure (–CH_2_) in the phospholipid molecule has characteristic absorption peaks in the infrared spectrum; by measuring the intensity and position of the characteristic absorption peak, the structure change and content of phospholipids can be accurately detected, as illustrated in Figure 3a. Daniel et al. utilize multiresonant mid-infrared metasurfaces to simultaneously provide up to three orders of magnitude local near-field intensity enhancement across the amide and methylene bands. As shown in Figure 3b, by combining real-time spectral acquisition with advanced linear regression analysis, different phospholipid molecules can be distinguished and the interaction kinetics can be accurately tracked. The sensor is ideally suited for real-time monitoring of the movement of lipid-protein systems in aqueous environments and studying a range of important processes such as lipid-protein binding, protein-induced membrane disruption, and vesicle cargo release (Figure 3c). By studying the interaction of the pore-forming toxin melittin with the lipid membrane, this method can independently track melittin and lipid signals and reveal the membrane disruption caused by melittin (Figure 3d). Furthermore, label-free real-time monitoring of neurotransmitter release from synaptic vesicle mimics demonstrates the applicability of this approach in biomolecular systems of increasing complexity. Thus, the sensor facilitates the study of important classes of lipid vesicles such as synaptic vesicles in neurodegenerative diseases, exosomes in cancer, and drug release mechanisms of liposomes in pharmaceutical research.

### 2.3 Cell detection

**Figure 4:**
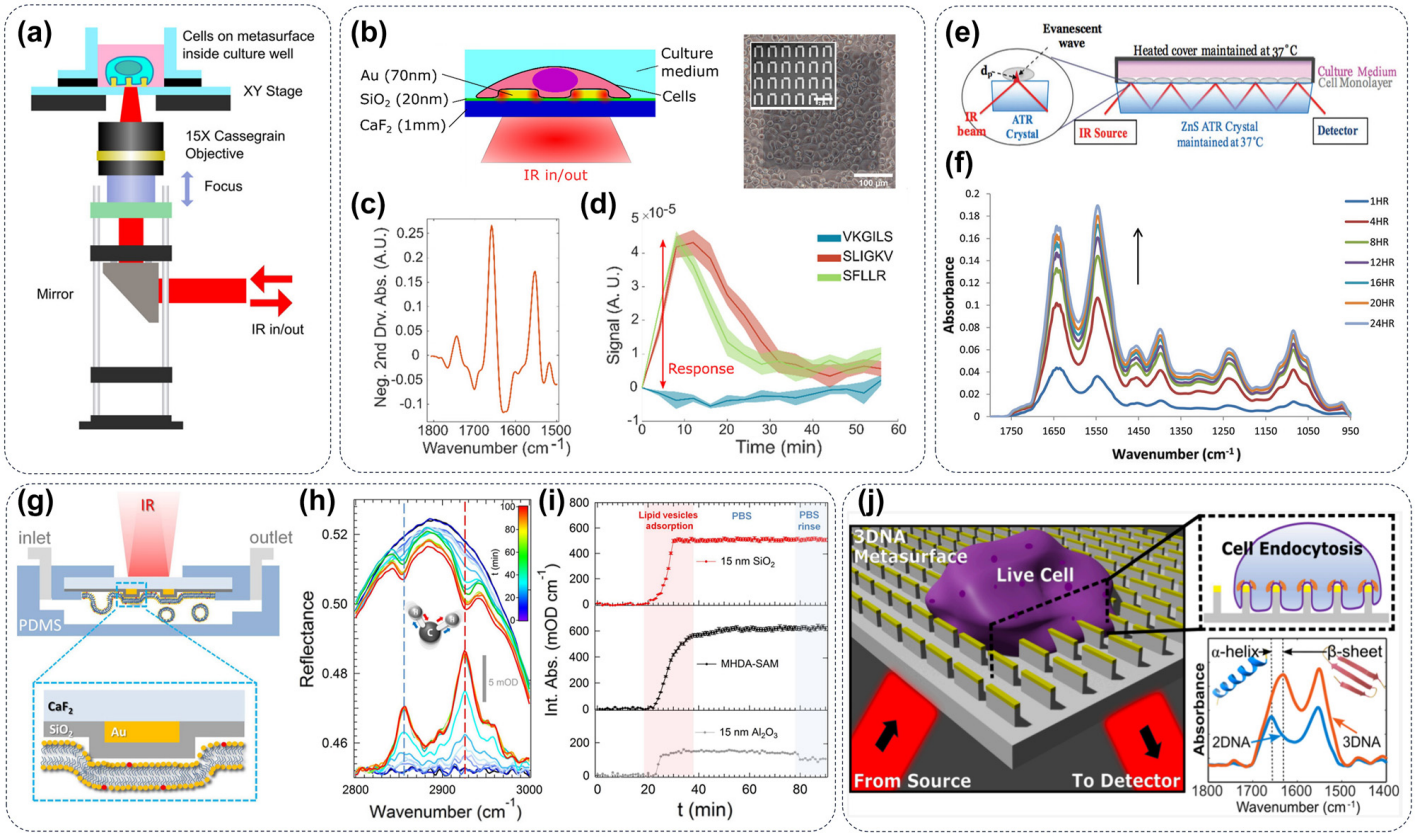
Metasurface for IR imaging of live cells. (a) Schematic drawing of the light path diagram for *in vivo* imaging of cells by infrared spectroscopic microscopy [72]. (b) Cells can efficiently adhere to the metasurface substrate, and (c) the signal amplification of the metasurface can facilitate the SEIRA measurement cellular response to (d) the activation of PAR1/PAR2 receptors by three synthetic peptides [72]. (e) A schematic diagram illustrates the multireflection ATR-FTIR measurement setup and (f) a representative set of ATR-FTIR spectra obtained from live MDA-MB-231 cells after a 24-h seeding period [73]. (g) A schematic representation depicting the fluidic chamber and experimental setup during measurements. The yellow and red phospholipid headgroups signify DOPC and Texas RED DHPE, respectively. (h) The vertical dashed lines indicate the spectral positions of the symmetric (depicted in blue) and asymmetric (depicted in red) CH_2_ stretching modes and (i) the significant differences in the adsorption efficiency of phospholipids on different materials [79]. (j) IR spectroscopic detection of protein (including clathrin and AP-2 complex) conformational changes during clathrin-mediated endocytosis assisted by metasurface [42].

Water molecules have strong attenuation of IR light, which will greatly lead to a low signal-to-noise ratio and prolong signal collection times, particularly hindering the detection of amide vibrations [71]. Hence, the experiments we discussed in the Sections 2.1 and 2.2 were conducted under atmospheric conditions that were not representative of the proteins’ native environment. In practice, the water content in biological samples such as cells and tissues is often higher than 70 %, the hydrogen bonds of water molecules and the polar environment of aqueous solution will participate in the change of protein secondary structure, and the observed results under atmospheric conditions may not reflect the true results, thereby impeding the application of resonant SEIRA to biologically relevant systems. Cell is the basic unit of organism metabolism, and the detection of cell function involves a series of substances such as nucleic acid, protein, and lipid, which has become an important research direction of infrared spectroscopy. The optical resonances of metasurfaces can be easily modulated in a wide frequency range, so that the infrared signal of the cell membrane protein can be amplified via metasurface, and the interference of the absorption of water molecules on the infrared detection can be suppressed, thereby realizing the infrared spectral analysis of biomolecules in an aqueous solution. As shown in Figure 4a, to avoid the strong adsorption of infrared light by a high abundance of water molecules in cell culture medium and cells, a series of detection methods for cell culture and signal methods based on metasurfaces were proposed for living cell detection. The utilization of plasmonic metasurface in conjunction with enhanced infrared spectroscopy is demonstrated in Figure 4b, showcasing its potential as a cellular analysis technique. As shown in Figure 4c and d, cell adhesion on metasurfaces with different surface coatings and cellular responses to activation of the protease-activated receptor (PAR) signaling pathway were characterized by changes in cellular infrared spectra [72]. In addition to transmission Fourier Transform Infrared Spectroscopy (FTIR), the ATR sampling technique has proven effective for investigating live cells. The sampling volume in ATR is constrained by its penetration depth, typically limited to approximately 2 μm above the surface where cells adhere to the ATR element. Results in Figure 4e indicated that attaching the cells on the surface of a single-bounce or a multi-bounce ATR crystal, and then the evanescent field of ATR of mid-infrared light at the cell/crystal interface was collected via FTIR microscope. With the ATR-FTIR methodology, the evanescent field effectively penetrates approximately 1–2 μm into the cell and its surrounding culture medium, the above penetration depth range contains a large number of signals from various organic substances in cells (Figure 4f), which avoids the interference of water absorption. The efficacy of ATR-FTIR as a cellular assay was demonstrated by measuring cellular adhesion and spreading on various ECM surface coatings [73]. With the advantage of highly sensitive detection of conformational changes in membrane proteins and support of deep learning, the ATR-FTIR approach has become a powerful tool for observing the interaction between drugs and membrane proteins. The combination of the living cell ATR-FTIR approach and high-throughput technology can provide effective support for large-scale drug screening [74], [75]. However, the ATR-FTIR approach is not compatible with existing commercial infrared microscopes, which are limited to reflective infrared detection. Consequently, researchers must manually construct a microscope system tailored to their research objectives. Furthermore, the effectiveness of the ATR-FTIR approach heavily relies on the alignment between the calibration model in the database and samples, thereby restricting its widespread application [76], [77], [78]. Although SEIRA has many limitations for live cell detection in aqueous solution, researchers have made great efforts to improve the elemental composition and spatial arrangement of metasurfaces and successfully applied SEIRA to infrared imaging of living cells. By harnessing the potent electromagnetic field enhancement facilitated by metasurfaces featuring tailored optical resonances (as depicted in Figure 4g), SEIRA effectively mitigates the inherent limitations of reflective IR detection signals. Subsequent investigations have demonstrated that these metasurfaces can remarkably amplify the IR signal within a spatial range of tens of nanometers above their surface. With the design and optimization of metasurfaces with metal-on-dielectric structural configuration, the researchers used a SEIRA microscope to achieve highly sensitive detection of the infrared spectral fingerprint information of lipid membranes and proteins within 100 nm above the metasurface (Figure 4h). More importantly, the findings depicted in Figure 4i demonstrate a direct correlation between the infrared signal intensity of lipids and the material composition of the metasurface, thereby suggesting potential interactions between specific lipid molecule groups and the metasurface [79]. In summary, numerous experiments have successfully demonstrated the infrared detection of proteins and lipids in solution, which are vital constituents of cells, leveraging the signal amplification capabilities offered by metasurfaces. Inspired by the above studies and the recent introduction of high-aspect-ratio nanostructures as a new platform for manipulating cell behavior, Aditya et al. demonstrate that the integration of plasmonic and dielectric nanostructures significantly enhances the cell testing capability of FTIR spectroscopy, as shown in Figure 4j [42]. The observation of protein translocation to the high curvature membrane region during cell adhesion was realized, which laid the foundation for the observation of cell physiological changes by infrared spectroscopy.

## 3 Metasurface-enhanced Raman spectroscopy

Raman spectroscopy is generated by inelastic light scattering via molecular vibration, which can provide fingerprint information for biological detection and medical diagnosis [80]. However, the inherent low scattering intensity of conventional Raman spectroscopy results in a very weak detection signal, which limits its wide application [81]. SPR modes in metal nanostructures can efficiently capture incident light due to their large effective cross section [82]. At resonant wavelength, a significant amplification of the incident optical field can be achieved from a collected area that far exceeds the physical size of the metallic nanostructures [83], [84]. Hence, plasmonic nanostructures are exceptionally beneficial in amplifying inherently weak optical processes, such as surface-enhanced Raman scattering (SERS) [85]. However, in biological detection, the tunability and reproducibility of hot spots within plasmonic nanostructures are crucial, posing significant challenges in nanofabrication [86]. The optical resonance of metasurfaces can be precisely tuned by manipulating their design parameters, such as size, dielectric environment, geometry, and material composition. These high degrees of design freedom and flexibility enable metasurfaces to meet all the requirements for being an ideal Raman substrate in biomedical detection [87], [88], [89].

**Figure 5:**
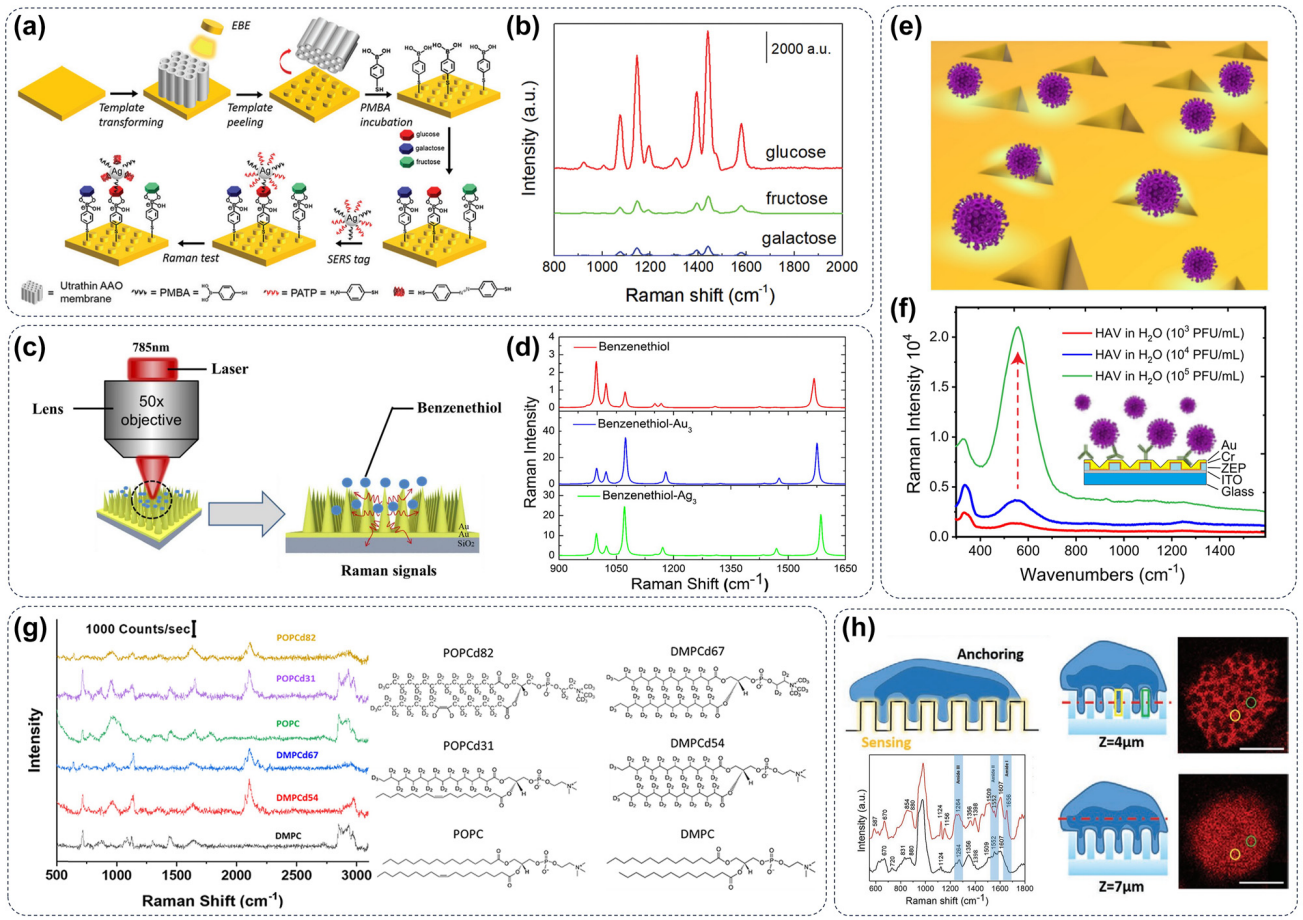
Metasurface for SERS imaging in biomolecules. (a) Schematic diagram of a classic metasurface structure for SERS detection and (b) its application in monosaccharide identification [43]. (c) Illustration of experimental advancements in SERS utilizing a microcone-based plasmonic metasurface for enhanced performance. (d) The elemental composition of the metasurface is optimized to obtain the strongest SERS signal [91]. (e) Schematic diagram of the inverted pyramid metasurface construction and (f) its application in label-free SERS technique for HAV virus concentration identification [92]. (g) Lipid adsorbed on a metasurface can be precisely analyzed for its molecular composition by SERS [93]. (h) A schematic representation of the nanopillar-based Raman optical detection platform facilitating deformation in the cell membrane and nucleus. This design enlarges the contact area between the metasurface and the cell and obtains a stronger SERS signal of the cell membrane [94].

As shown in Figure 5a and b, glucose, fructose, and galactose are common types of hexoses (C_6_H_12_O_6_) that have the same molecular weight, so they cannot be distinguished by traditional analytical chemistry methods such as mass spectrometry. However, the chemical structures (or chemical bonds) of three hexoses are significantly different, which can be adsorbed on the surface of the metasurface by phenomenological acid modification [43]. By exploiting the SERS effect facilitated by metasurfaces, the identification of hexoses is accomplished based on discerning discrepancies in their respective Raman spectra. It has become an important means to decrease the limit of detection (LOD) of Raman signals of bioactive small molecules by achieving guide light in the nanoscale, thereby improving the enhancement factor (EF) of the metasurface to Raman signals [90]. Meanwhile, Figure 5c illustrated a plasmonic metasurface for benzenethiol detection, which uses gold micro-cone arrays to excite SPR waves propagating along the conical tip toward the apex region, thus increasing the local strength of the electromagnetic field by several orders of magnitude. The metasurface composed of gold element showed the strongest SERS signal as shown in Figure 5d, which exhibits a remarkable LOD of 10^−8^ mol/L and an EF of 10^9^ by possessing the capability for SERS measurement [91]. In addition to the optical field control, the hydrophilic and element composition of the substrate are also very important for the application of metasurfaces in SERS-based biological detection. The metasurface shown in Figure 5e is composed of gold or silver easily adsorbs biomolecules such as antibodies, and then the antibody can be further used to capture the HAV virus in the body fluid and realize high sensitivity detection of the virus via SERS detection (Figure 5f) [92]. Encouraged by the outstanding Raman signal enhancement capabilities of metasurfaces for small molecules, researchers have demonstrated that metasurfaces can also be applied to SERS detection of complex structures such as cells. The cell membrane is composed of a phospholipid bilayer and different ratios of phospholipids will endow the phospholipid bilayer with different fluidity, which is convenient for the cell membrane to perform different functions. For example, liposomes are widely used as drug delivery vehicles, and different phospholipid compositions will also affect their delivery efficiency and intracellular delivery areas. Considering that SERS can detect changes in chemical bonds, thereby enhancing our understanding of the dynamics and interactions during liposome delivery, as illustrated in Figure 5g, Bruzas et al. reported a novel silica-coated silver film-over-nanosphere substrate to successfully create a SERS-active lipid bilayer identification system [93]. Encouraged by this, some label-free SERS detection of specific cell surface markers in living cells based on metasurfaces has shown great practicability. For instance, accurate identification of the expression level differences in prostate-specific membrane antigen expression on the cell membrane of prostate cancer cells with the same phenotype has been realized, as shown in Figure 5h [94].

**Figure 6:**
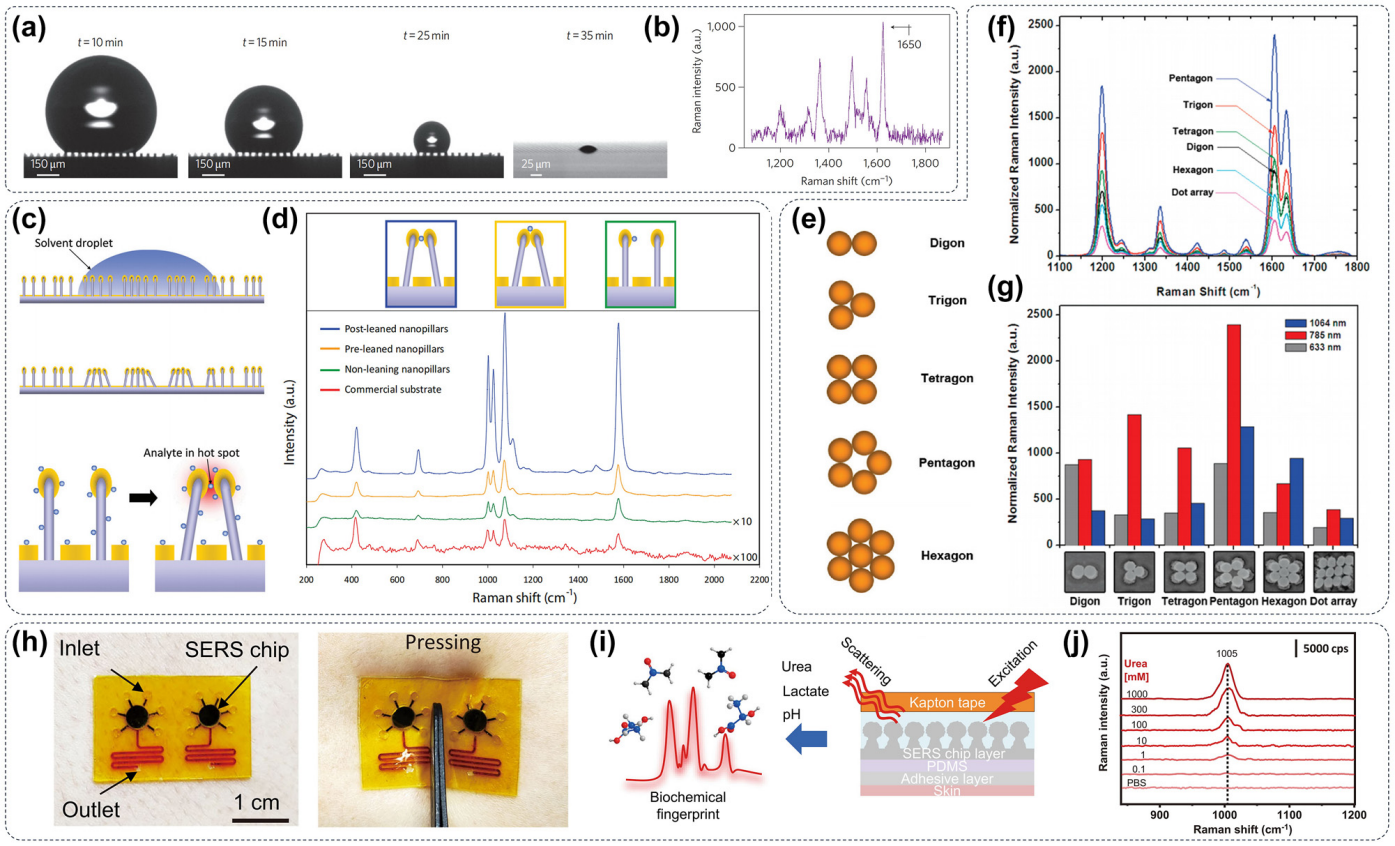
Flexible metasurface for SERS detection. (a) During the volatilization process, the droplet is gradually condensed to a very small area on the metasurface via surface tension and (b) the high concentration of the residual substance in the liquid improves the signal intensity of SERS [96]. (c) The scheme depicts the process of droplet evaporation, and the surface tension of the aqueous solution promotes the formation of new hot spots on the flexible metasurface structure and enhances the intensity of the SERS signal. (d) The Raman spectra revealed that the inclined nanopillars exhibit significantly greater Raman enhancement compared to the noninclined nanopillars [97]. (e) Diagrammatic representations of polygonal assemblies using nanospheres for the different shapes. (f) Normalized Raman spectra of *trans*-1,2-bis (4-pyridyl)-ethylene for different shapes. (g) Comparison of the normalized intensity of the Raman signal at 1,600 cm^−1^ excited at 633, 785, and 1,064 nm [99]. (h) The as-prepared microfluidic SERS sensor attached to the skin exhibits good flexibility. (i) Schematic of multi-SERS analysis based on the microfluidic plasmonic device. (j) SERS spectra of different concentrations of urea in a label-free manner [103].

Detecting trace molecules in aqueous solution is of great significance in the fields of biomedicine and ecological pollution. However, plasmonic metasurface sensors cannot be directly used to detect molecules dissolved in femtomole or atom mole solutions due to the limited detection sensitivity [95]. To overcome this limitation, Figure 6a indicated that combining superhydrophobic artificial surface and nano-plasma structure to bind droplets on the metasurface and volatilize them to increase the concentration of the substances to be detected and then combined with SERS to achieve detection of the chemical structure of trace specific molecules (Figure 6b) [96]. During the volatilization of water droplets, the surface tension of water will induce the controllable aggregation of flexible components in the metasurface as illustrated in Figure 6c, thus generating new hot spots to further improve the enhancement of SERS signals by metasurface materials (Figure 6d) [97]. The hybrid approach of integrating top-down fabrication techniques with self-assembly processes facilitates the formation of complex nanoplasmonic structures with sub-nanometer gaps between gold nanoparticles [98]. Combining the theoretical simulation with the experimental results as shown in Figure 6e, the symmetry of the nanoplasmonic structures was analyzed specifically for their application by SERS, revealing that the pentagonal assembly exhibited the highest degree of Raman enhancement for the molecules being tested in Figure 6f and g [99]; this result can further guide the design strategy of flexible metasurfaces to obtain stronger SERS signals [100]. Since the components of the metasurface can be flexible, can the whole metasurface be designed to be flexible? The butterfly wings look colorful because they are covered with numerous nanoscale structures, which can produce physical phenomena such as refraction, reflection, and interference of light, and can be considered as a natural meta-structure [101]. Hence, the flexible nanostructures of butterfly wings have inspired researchers to design fully flexible metasurfaces [102]. He et al. designed a miniature, thin plasmonic metasurface with homogeneous mushroom-shaped hot spots and high SERS activity integrated into a flexible microfluidics platform as illustrated in Figure 6h, which realized the real-time monitoring of metabolites in human sweat (including urea, lactate, and pH) as shown in Figure 6i and j, and provided a controllable, convenient, and dynamic biofluid sensing system for personalized medicine [103].

## 4 Metasurface-enhanced fluorescence spectroscopy

Fluorescence spectroscopy is one of the most commonly used methods in the field of biomedical detection, which has the advantages of high sensitivity, fast responsiveness, and quantitative analysis [104], [105], [106], [107]. Commonly used fluorescence detection instruments, including fluorescence spectrometer, living cell fluorescence microscope, fluorescence confocal microscope, quantitative real-time PCR, and so on, have almost become the necessary tools for biomedical researchers [108], [109], [110], [111]. However, when fluorescence spectroscopy is applied to liquid biopsy, such as the detection of exosomes, miRNA, or protein disease markers in serum, it is difficult to obtain high signal-to-noise ratio detection results because the concentration of these disease markers is often less than 10^−10^ mol/L, which is close to the LOD of the above methods [112], [113], [114]. To further improve the sensitivity and reduce the LOD, it is necessary to find excellent platforms for fluorescence sensing [115]. Metasurfaces have recently emerged as powerful platforms for enhancing fluorescence sensing, which can achieve thousands-fold fluorescence intensity enhancement, and the LOD fluorescence spectrum can reduce to sub fmol/L [116], [117]. Another way to reduce the LOD is to build advanced optical imaging systems. This strategy also faces significant challenges, particularly in terms of integrating bulky and cumbersome optical components within a limited space, and the confined area may severely limit the space available for positioning and manipulating bio-specimens. Integrating metasurface into the fluorescence imaging system indeed presents a promising approach to effectively address this problem since they can efficiently modulate both the amplitude and phase of light [118], [119]. Leveraging their capability to manipulate light at the nanoscale, metasurface offers an exceptional platform for developing planar photonic devices tailored to specific functionalities [116], [120], [121]. Next, we will describe the application of metasurface in fluorescence spectroscopy in detail.

### 4.1 Reducing the LOD in fluorescence detection

**Figure 7:**
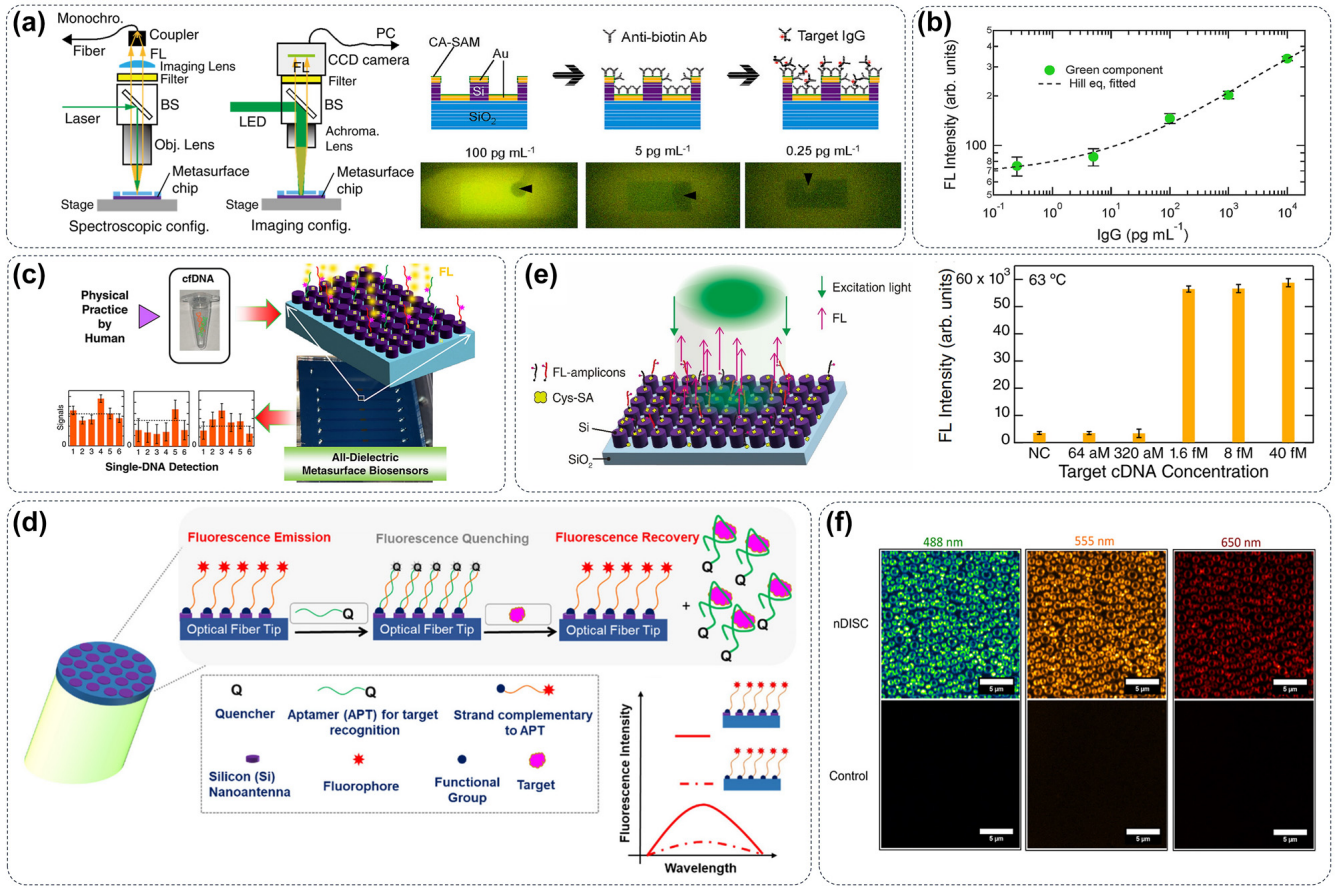
Metasurface materials can enhance fluorescence signals. (a) Light path diagram of classical fluorescence spectrum detection and schematic diagram of metasurface structure applied to fluorescence signal enhancement [122]. (b) The fluorescence enhancement effect is mediated by the metasurface material to realize the linear detection of the concentration of disease markers at the picogram level [123]. (c) The metasurface controlled the self-assembly of DNA probes on the surface, thus realizing the trace detection of cfDNA-related disease markers [124]. (d) Through the precise regulation of fluorescence quenching and enhancement, the metasurface structure can realize the synchronous detection of nucleic acid and protein [125]. (e) The metasurface structure can assist fluorescence spectroscopy to quickly realize attomolar detection of SARS-Cov-2 [126]. (f) A broadband fluorescence-enhancement factor of Al-based metasurface can realize the detection of a plurality of disease markers (in different fluorescence channel) at the same time [44].

Metasurfaces can be conveniently embedded in the commonly used fluorescence detection state, such as the well plate, glass slide, or cell culture dish, which has extremely wide application in the aspect of biomedical detection. Under the enhancement effect of the metasurface on fluorescence signals as illustrated in Figure 7a, the detection of pg-level disease markers can be realized, which is far lower than the LOD of traditional fluorescence detection methods [122]. Accurate detection of trace disease markers in serum is very important for early diagnosis and treatment, Iwanaga et al. developed a highly sensitive fluorescence biosensor by integrating gold nanostructures with hybrid silicon waveguides. This innovative design enabled the detection of ultra-low concentrations of IgG antibodies down to just 5 pg/mL (34 fmol/L), as illustrated in Figure 7b [123]. In addition, the detection of free micro-RNA or cell-free DNA (cfDNA) in serum is also of great significance for the early detection of tumors and other diseases. Some studies indicated that combining high-fluorescence all-dielectric metasurface biosensor with a short-time nucleic acid amplification technique can detect single-target cfDNA with high precision, thereby realizing the test distinguishes 1 copy/test from zero, unachievable by techniques like digital PCR (Figure 7c) [124]. Based on the above research, Figure 7d depicts an aptamer-based displacement assay that labeled aptamers on the metasurface for target molecules detecting dissolved in solution [125]. Specifically, when the labeled aptamer strand binds to the metasurface and hybridizes with a recognition aptamer that contains a quencher, the quencher moves close enough to cause fluorescence quenching. When the target molecule is present in the solution, the aptamer forms a strong complex with the target molecule, and then the quencher and fluorophore are separated. This separation leads to a recovery of fluorescence emission that correlates with the target concentration. The presence of metasurface on the substrate enhances the fluorescence intensity, enabling the detection of targets even at very low concentrations in the solution. The fluorescence enhancement strategy based on metasurfaces can achieve short-time and extremely high sensitivity detection, relevant studies have shown a metasurface biosensors successfully detected amplicons derived from attomolar concentrations of SARS-CoV-2 nucleic acids, and the amplification process was completed within 1 h, meeting the criterion for reliable infection tests, as illustrated in Figure 7e [126]. Many chronic diseases need a combined diagnosis of multiple markers to better guide the identification of diseases and the establishment of follow-up treatment programs, it is imperative to develop metasurfaces capable of effectively enhancing multichannel fluorescence. Metasurfaces comprising Al nanoantennas of nanodisks-in-cavities possessed the capability to achieve substantial enhancement of both near-field and far-field interactions in the visible spectrum, thereby facilitating intense and broadband excitation and emission of fluorophores. By synchronously amplifying fluorescent signals generated by a plurality of fluorophores, a plurality of markers can be simultaneously detected in different fluorescence channels such as 488 nm, 555 nm, and 650 nm (Figure 7f) [44].

### 4.2 Improving the detection capability of existing fluorescence microscopy

**Figure 8:**
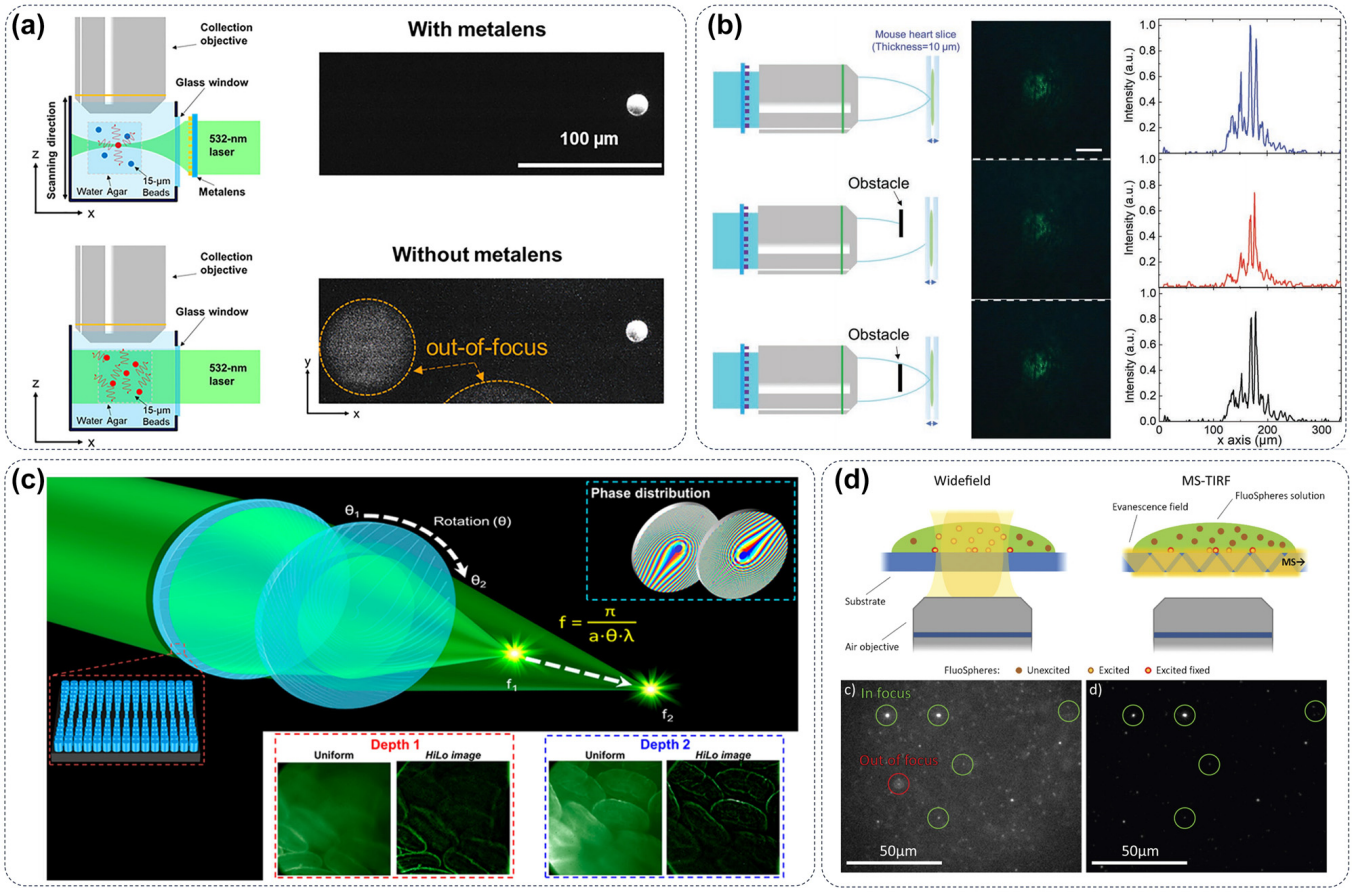
Metasurface improves the imaging ability of fluorescence microscopy. (a) The spatial resolution of a fluorescence microscope can be improved by embedding a GaN metasurface into the optical path of the microscope [127]. (b) Metasurface structures generate an abrupt autofocusing beam to improve the deep tissue imageability of fluorescence microscope [128]. (c) The moiré metalens consists of two complementary phase metasurfaces that eliminate the spherical aberration and distortion generated in the process of fluorescence microscope imaging [129]. (d) The metasurface facilitated the coupling of light into a microscopy coverslip, enabling the achievement of TIR excitation [130].

Light-sheet fluorescent microscopy (LSFM) stands as the preeminent technique used *in vivo* fluorescent images for disease research, medical science, and cellular biology. However, crafting an illumination system that guarantees high image resolution and optical sectioning poses a significant challenge, and the bulky components required for illumination and detection introduce geometric constraints. Hence, integrating a nanophotonic metasurface as the illumination element in LSFM can effectively tackle these issues. The metalenses composed of 800-nm-thick gallium nitride (GaN) nanostructures are tailored to produce a light sheet that is ideally suited for imaging biological samples. By incorporating the metalenses, the complexity of the LSFM system was significantly streamlined, enabling to capture of multicolor fluorescent images of live *Caenorhabditis elegans* with cellular-level resolution, which paved the way for acquiring high-resolution *in vivo* images of biological specimens shown in Figure 8a [127]. One of the significant limitations of fluorescence detection *in vivo* is the limited penetration depth of fluorescence. Metasurfaces possess the ability to manipulate light properties at the nanoscale, allowing for precise control over input beam parameters. This advantage enables the creation of an abrupt autofocusing (AAF) beam through a nanophotonic metasurface, which holds great promise for various biomedical applications. The utilization of fluorescence-guided laser microprofiling in mouse cardiac samples has been investigated through experimental studies, as depicted in Figure 8b. In this approach, the AAF beam is employed to precisely deliver optical energy to targeted locations [128]. Meanwhile, tunable lenses in microscopy acquire multiplane images but suffer from spherical aberration and distortions. The moiré metalens with variable focal length (10–125 mm at 532 nm laser wavelength) addresses this by tuning mutual angles of two complementary phase metasurfaces. In addition, a telecentric configuration using the moiré metalens to ensure high-contrast multiplane fluorescence imaging for *ex vivo* mouse intestine samples shown in Figure 8c [129]. The above findings highlight the significant potential for integrating metasurface optics in the development of compact laser surgery instruments, which could have broad applications in the field of biomedicine.

Recently, significant advancements in dielectric metasurfaces for visible light have resulted in the development of ultra-thin optical devices that enable precise manipulation of optical wavefronts with a remarkable degree of freedom. The metasurface can couple light into a microscopy coverslip for total internal reflection (TIR) excitation in TIR fluorescence microscopy (TIRFM). Traditional objective-type TIRFM requires bulky, expensive objectives with limited field-of-view (FOV). The metasurface made of periodic planar TiO_2_ nanostructures on borosilicate glass achieves high-efficiency coupling under oblique incidence with an incident angle of 65°, minimizing background fluorescence, and achieving immunostained imaging of human mesenchymal stem cells over a FOV of 200 µm × 200 µm as illustrated in Figure 8d [130]. Overall, metasurface promises low-cost, high-contrast, low-photodamage TIRFM with high surface selectivity.

## 5 Metasurface-enhanced other spectral modalities

### 5.1 Colorimetric biosensor

**Figure 9:**
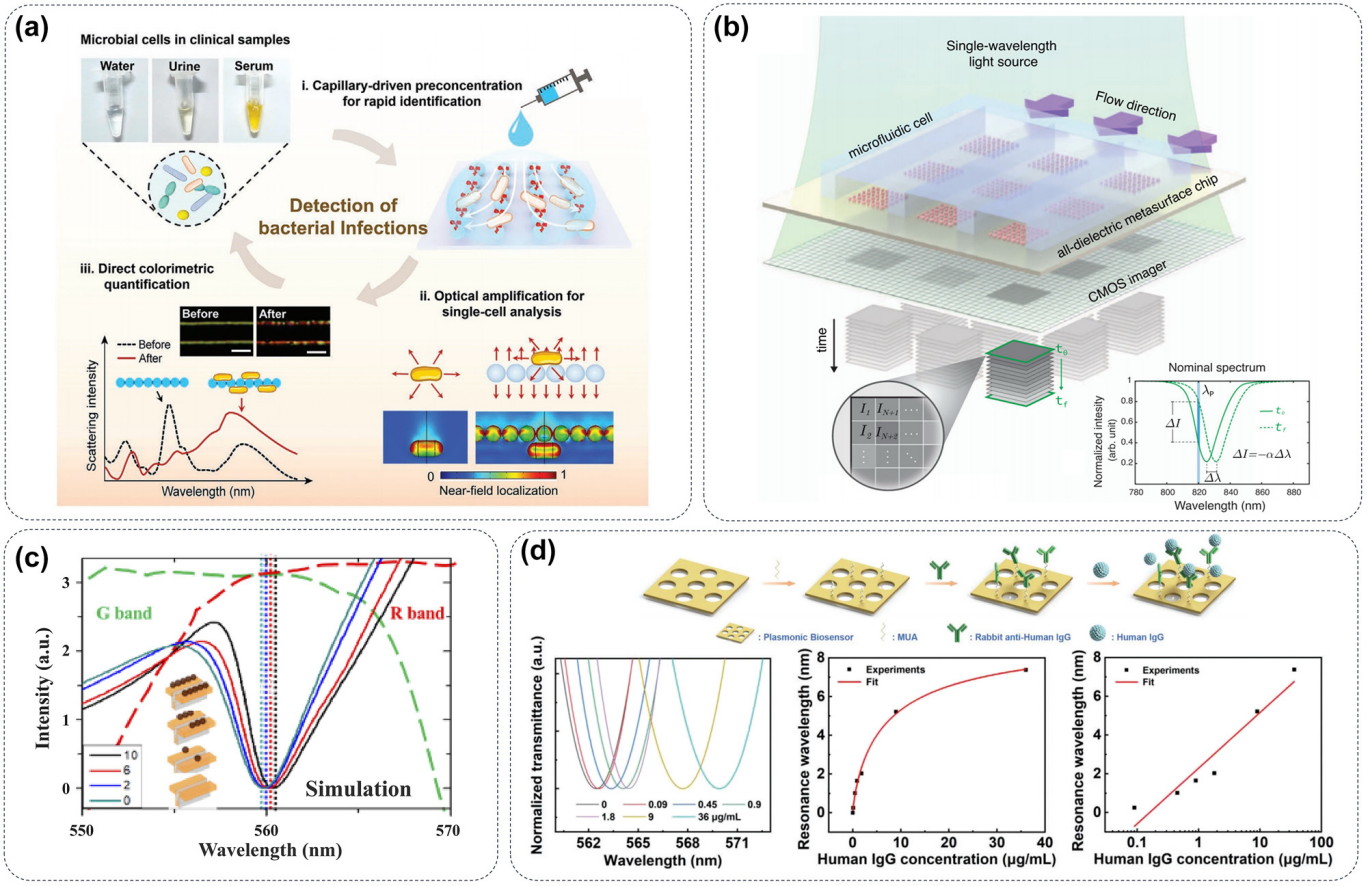
Colorimetric biosensor of metasurface. (a) A novel label-free, ultrasensitive, and visual biosensing technology is developed based on a printed nanophotonic metasurface [131]. (b) Large-area metasurface chips, configured as microarrays and integrated with microfluidics on an imaging platform, enable real-time detection of breast cancer extracellular vesicles [136]. (c) The color images of nanoplasmonic sensing metasurface have been developed for sensitive and label-free detection of virus-like particles [139]. (d) Metasurface constructed colorimetric detection system can achieve a logarithmic fit detection of 0.09–36 μg/mL of the human IgG [140].

Metasurface-modified biochip is a widely used colorimetric analysis tool, which can quickly read out the content of analysis according to the color change of the refracted light on the metasurface [45]. Upon adsorption of biomolecules onto the metasurfaces, a slight color change is observed in the reflected image of the system due to the interaction between the resonant-induced near-field enhancement via metasurfaces and the biomolecules. With the large adjustability of resonant wavelength for the metasurface that can amplify the weak optical signal, the colorimetric detection result can be visually detected by a traditional optical microscope or a portable mobile phone, which greatly improves the portability of the detection. Zhang et al. have realized the trace detection of a series of disease markers such as nucleic acid, protein, exosomes, viruses, and bacteria by metasurface structure with the characteristics of easy chemical modification and adjustable resonance absorption wavelength shown in Figure 9a [131], [132], [133], [134], [135]. These works provide a versatile optical platform for the detection and sensing of nanoscale disease markers, which can be widely used in biochemical detection and medical fields. To further simplify the detection system, researchers integrated dielectric metasurfaces with hyperspectral imaging to create an ultrasensitive, label-free biosensing platform as shown in Figure 9b. The technique captures spatially resolved spectra from millions of pixels and extracts high-throughput sensing data at an average of 0.41 nanoparticle/μm^2^. Unique sensor design allows spectral data retrieval from a single image, enabling portable diagnostics, which fusion of nanophotonics and imaging optics broadens the scope of dielectric metasurfaces for analyzing biological entities and two-dimensional materials over vast areas [136], [137]. As a common element for the preparation of metasurfaces, gold has the advantages of a strong SPR effect in the visible light region, easy chemical modification, and high reflectivity, which is a very suitable material for colorimetric biosensors [138]. Li et al. constructed a metasurface composed of gold nanostructures that can achieve the LOD of less than 1 pg/mL for the SARS-CoV-2 virus in less than 30 min, as illustrated in Figure 9c [139]. At last, the cost-effective fabrication of plasmonic metasurfaces is a key factor for biomedical research. To combine nano-imprint lithography, electrodeposition, and nanotransfer printing to create nanoscale resolution templates, the centimeter-scale metasurfaces can quickly fabricate with uniform gold nanopillars. Moreover, to examine the effectiveness of the grafting process and gain insights into the dynamic modifications involving EDC-NHS and the coupling processes of IgG/anti-IgG during the experiments, the shift of the resonance wavelength in the transmission spectrum can be monitored in real-time. As shown in Figure 9d, this design performs comparably to conventionally made metasurfaces in refractometric sensing and IgG detection, and the templates are reusable over ten times ensuring consistent performance [140].

### 5.2 SPR biosensors

**Figure 10:**
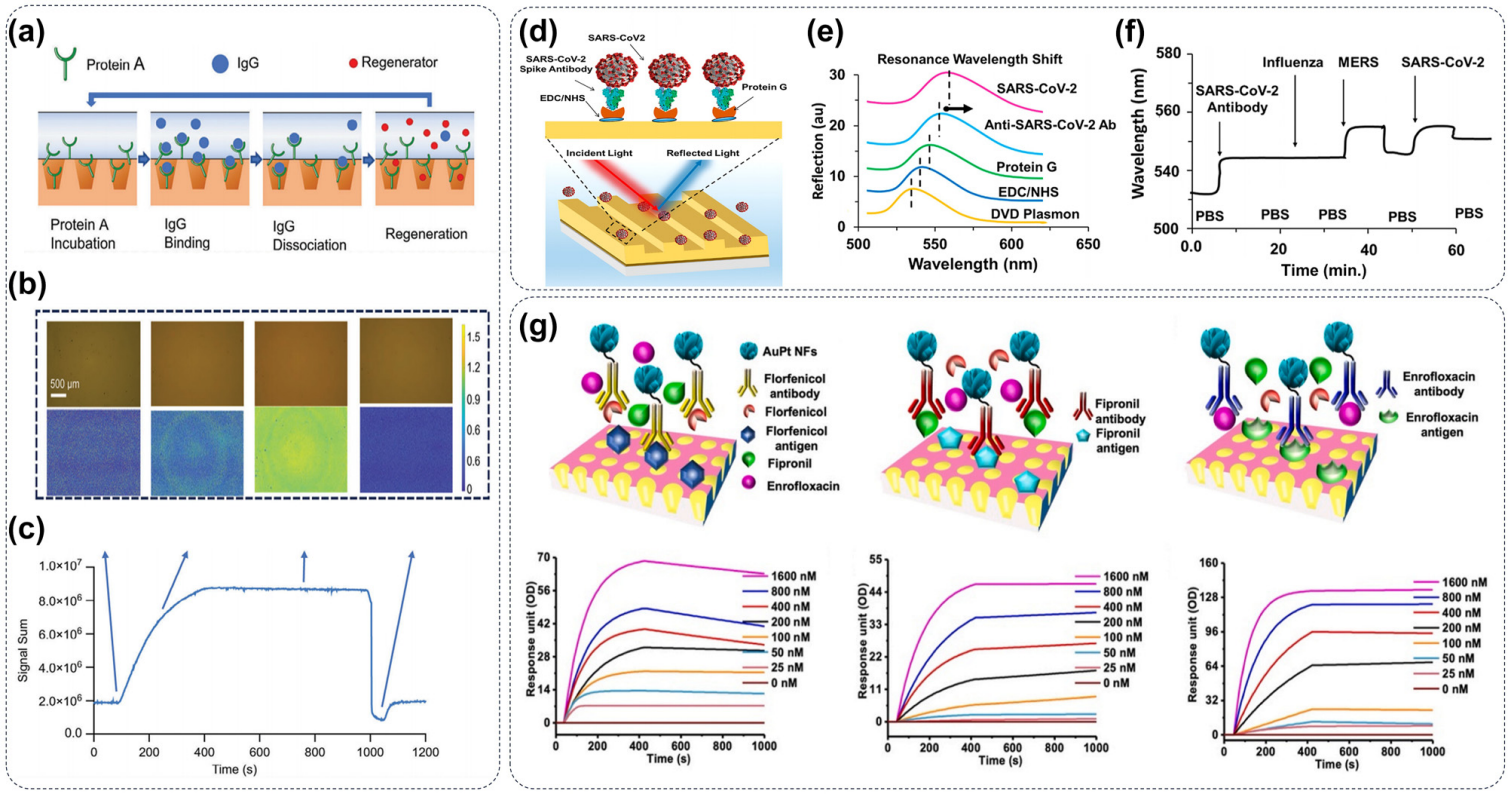
SPR biosensors of metasurface. (a) A graphical representation of the protein A-IgG kinetic experiment outlines four distinct phases: baseline, binding, dissociation, and regeneration. (b) Representative images illustrate the four phases of the experiment along with their corresponding signals. (c) A standard graphical representation shows the signal sum and the associated images [148]. (d) Schematic diagram of SPR-based metasurface for SARS-CoV-2 detection. (e) The operational principle of the proposed sensor relies on shifts in resonance wavelength resulting from molecular binding events, (f) enabling the differential identification of SARS, MERS, and influenza based on both specific and nonspecific interactions [149]. (g) A biosensor based on gold-platinum nanoflowers coupled with metasurface plasmon resonance is designed for multiplexed real-time detection of small molecules, such as florfenicol, fipronil, and enrofloxacin [150].

SPR sensors can detect the interaction between biomolecules without labeling, avoiding the false positive results caused by radioactive or fluorescent labels [138]. Moreover, SPR technology has extremely high sensitivity and can detect small changes in refractive index [141]. This allows it to detect very low concentrations of molecules and weak interactions, thus improving the accuracy and reliability of biological detection [142], [143], [144]. More importantly, SPR imaging technology can detect multiple samples at the same time, which improves the efficiency and throughput of the experiment [145], [146], [147]. The integration of plasmonic metasurfaces into an imaging system, as illustrated in Figure 10a, enables localized SPR sensing with microfluidic systems and bright-field imaging. This integrated system facilitates the accurate detection of concentrations ranging from 100 pmol/L to 100 nmol/L, while effectively removing nonspecific signals in real-time. Additionally, it allows for the automated measurement of kinetic curves at ten concentration gradients or simultaneous detection of multiple samples (Figure 10b and c). Consequently, this innovative approach offers cost-effective and convenient capabilities for detection [148]. The specificity of detection results can be enhanced by customizing the metasurface based on the characteristics of detected molecules, leading to a reduction in LOD. Rajib et al. use optical disc-based metasurfaces to excite asymmetric plasmonic modes, enabling tunable optical Fano resonance for multiple target detection in the visible range, as shown in Figure 10d. Through surface functionalization, the plasmonic metasurfaces facilitate real-time biosensing and demonstrated high-sensitivity, high-specificity detection of antibodies, proteins, and SARS-CoV-2 viral particles, distinguishing them from similar RNA viruses (Figure 10e and f) [149]. Metasurfaces can not only improve the specificity of SPR detection but also help to develop multiplexed small molecular detection methods. The metasurface immunoassays depicted in Figure 10g were developed as a one-step competitive assay, designed for integration into either a novel or standard plate device to enable multiplexed identification of small molecules (such as fluofenical, fipronil, and enrofloxacin) within a single egg. This innovative approach offers an impressive sensitivity enhancement of up to 1,000-fold and reduces the analysis time by 15-fold compared to conventional methods. Importantly, the results obtained from this technique exhibit excellent correlation with those obtained using liquid chromatography-tandem mass spectrometry [150].

### 5.3 Terahertz (THz) biosensors

The vibrational and rotational frequencies of many biological macromolecules are in the THz band, rendering THz spectroscopy highly suitable for the analysis of biomolecular interactions. This technique enables investigations into protein conformational changes, intermolecular interactions, and related phenomena [151], [152], [153], [154], [155], [156]. Meanwhile, THz imaging technology can provide fingerprint information of specific biomolecules at different levels, such as tissues, cells, and biomacromolecules, which can accurately distinguish different biomolecules or biological samples [157], [158], [159], [160].

**Figure 11:**
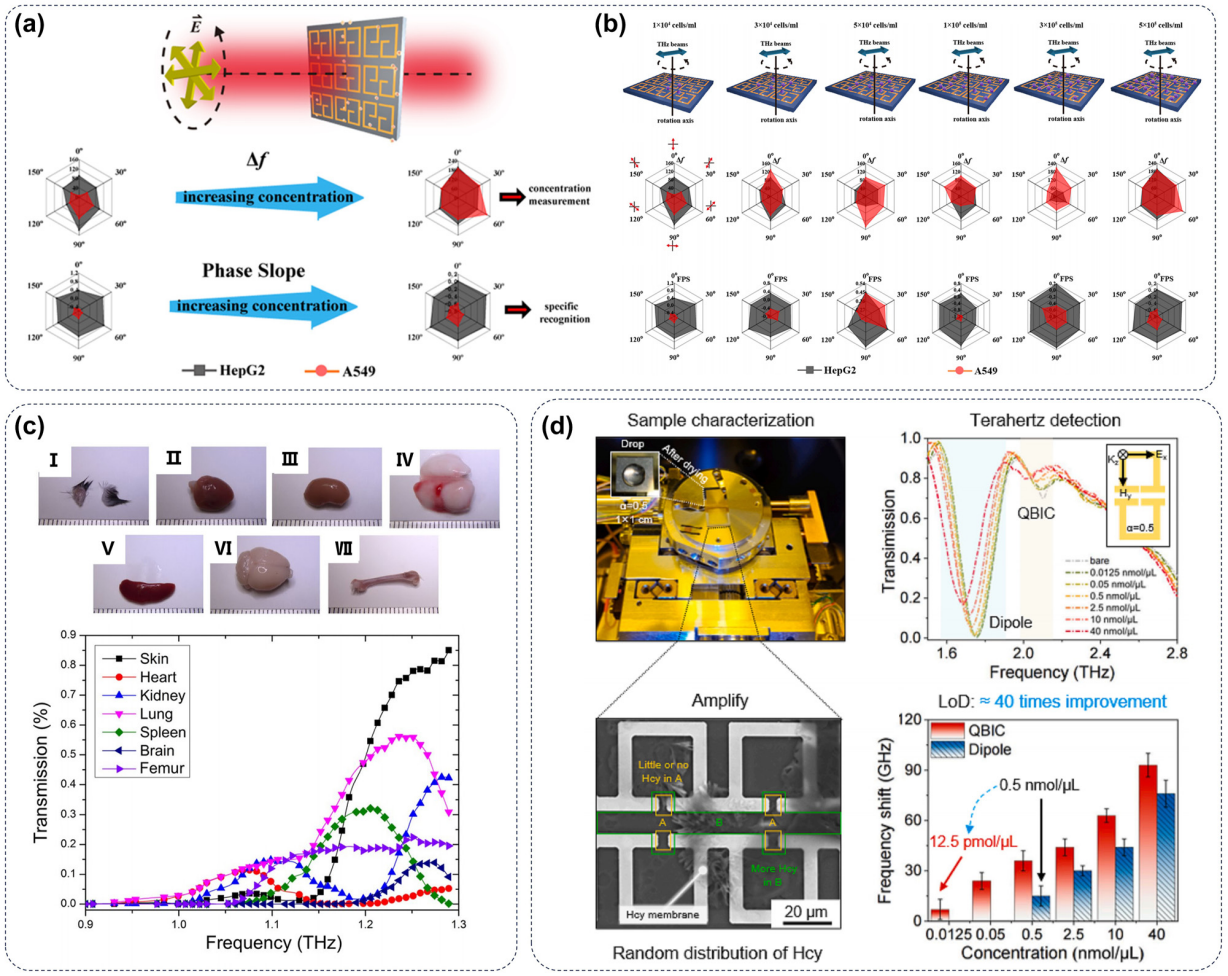
THz biosensors of metasurface. (a) Schematic representation of metasurface biosensor platforms: THz waves penetrating devices with cultured cancer cells. (b) The various types of cells can be distinguished by the difference of Δ*f* and fitted phase slope after being incubated on the substrate surface formed by the metasurface and irradiated by the THz sensor [161]. (c) The significant differences in THz absorption spectra of different mice organs [162]. (d) The qBIC within metasurfaces presents a viable platform for achieving robust in-plane light–matter interactions and offers promising prospects for the development of highly sensitive biosensors. Based on the statistical analysis, frequency shifts were extracted and compared between qBIC resonance and dipole resonance, revealing a direct LOD of 12.5 pmol/μL for qBIC resonance [163].

Figure 11a presents a nonimmune biosensing technology using plasmonic biosensors based on anisotropic resonators in the THz range to achieve antibody-free cancer cell recognition. By analyzing the frequency shift Δ*f* and the phase slope on cancer cell concentration at different polarizations, the lung (A549) and liver (HepG2) cancer cells can be distinguished via hexagonal radar maps. This technology can identify cells at concentrations as low as 10^4^ cells/mL and is valid over a wide range (10^4^–10^5^), as shown in Figure 11b. In addition, the two-dimensional extinction intensity cards based on wavelet transform can also provide various information for cell recognition. The plasmonic biosensors show potential for label-free cancer cell determination and recognition, serving as a nonimmune biosensing alternative [161]. Moreover, THz imaging can identify the distribution of different cells in organs because of the great difference in THz transmittance of cells (Figure 11c). Deng et al. have designed and fabricated a spiral-shaped plasmonic structure capable of achieving continuous frequency-tunable evanescent-field concentration within the THz range through simple operational means. Additionally, this device enhances the electric field intensity at the subwavelength aperture, resulting in a substantial increase in transmission. By merely rotating the spiral plasmonic structure, highly tunable transmission bands are observed, which align well with the anticipated behavior from electromagnetic simulations. Hence, this design combines plasmonic and frequency-tuning benefits, enhancing THz sensing and biomedical analysis [162]. In addition to *in vivo* detection, THz biosensors can also achieve highly sensitive detection of small molecules. An asymmetric metallic metasurface can support a q-BIC resonance with ultrahigh quality factor boosting light–matter interaction by 400 % in area and 1,300 % in intensity. Simulations and experiments show a high refractive index sensitivity of 420 GHz/RIU and a trace LOD of 12.5 pmol/μL for homocysteine. As shown in Figure 11d, this result offers a rapid, precise sensing method with potential in biochemistry, photocatalysis, and photobiomodulation [163].

### 5.4 Chiral biosensors

**Figure 12:**
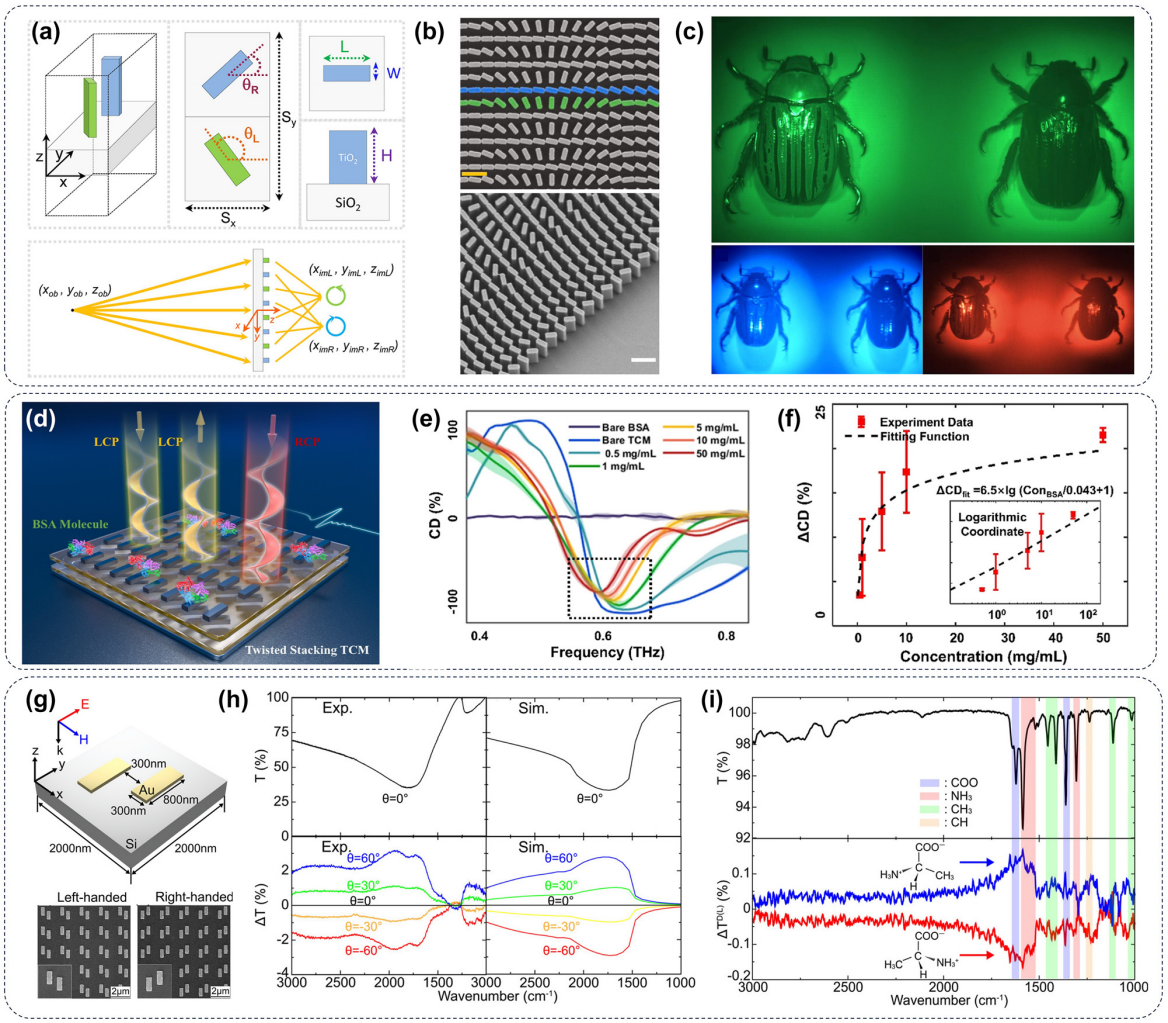
Chiral biosensors of metasurface. (a) Design and fabrication of the multispectral chiral lens (MCHL). The lower schematic diagram is provided to explain the imaging principle of MCHL where LCP and RCP light from the same object. (b) The top and side views of the metasurface structure. Scale bar: 600 nm. (c) The beetle imaging with MCHL [168]. (d) A schematic representation of the effortlessly crafted twisted stacking TCM designed for rapid biosensing. (e) Rapid detection of the concentration of BSA in solution via the enhancement of TCM. (f) Relation between ΔCD with the different concentrations of BSA solution. Insets denote responses plotted in logarithmic coordinates [169]. (g) Schematic and SEM pictures for design and fabrication of the gold nanorod-formed metesurfaces. (h) IR characterization of the metasurface. (i) Distinguish between D- (red) and L-alanine (blue) by super-chiral vibrational spectroscopy in the metasurfaces [170].

Circular dichroism (CD) spectroscopy can be used to detect and distinguish chiral substances, such as proteins, peptides, nucleic acids, and other biological macromolecules, which can reveal the fine structural differences of biological molecules [164], [165]. Moreover, CD spectroscopy is particularly important for the detection of trace chiral drugs and metabolites in organisms and has been widely used in biochemistry, pharmaceutical research, material science, environmental monitoring, and other fields [166], [167]. Figure 12a and b introduced aplanar metalens with an engineered dispersive response that creates two images with opposite helicity in one view, overcoming that traditional chiroptical techniques need complex optical components. Just with the lens and a camera, chiroptical properties can be analyzed across the visible spectrum without extra polarizers or dispersive devices. Researchers mapped the CD of a chiral beetle’s exoskeleton with high spatial resolution using this metasurface shown in Figure 12c [168]. More importantly, CD spectroscopy can not only identify insects but also is the best way to characterize chiral biomacromolecules. Figure 12d proposes a twisted stacking carbon-based THz chiral metasurface (TCM) using laser-induced graphene (LIG). This TCM achieves a high CD of +99.5 to −99.6 % in the THz band and demonstrates enhanced biosensing sensitivity of bovine serum albumin with a wide range (0.5–50 mg/mL) as illustrated in Figure 12e, and a nearly linear relationship can be achieved when the data are plotted on logarithmic coordinates (Figure 12f). This LIG-based TCM offers an eco-friendly platform for chiral research and has potential in low-cost commercial biosensing [169]. Recent advancements in plasmonic metasurfaces enable high-sensitivity vibrational CD spectroscopy of small chiral molecules like alanine enantiomers. Under linearly polarized illumination, a metasurface comprising an ordered array of staggered gold nanorods (depicted in Figure 12g) exhibits the capability to selectively generate left- and right-handed super-chiral fields at a wavelength of 1,600 cm^−1^, as illustrated in Figure 12h. This wavelength spectrally overlaps with the functional group vibrations of alanine. By harnessing these super-chiral fields, the experimental results depicted in Figure 12i successfully demonstrate distinct CD spectra of D- and L-alanine under linearly polarized excitation. Numerical simulations elucidate the resonant chiroptical interaction, establishing a robust chiral sensing platform for advanced infrared inspection [170].

## 6 Outlook and conclusions

This review discusses recent advancements in the detection of various biomarkers using metasurface-enhanced infrared absorption, Raman scattering, and fluorescence spectroscopy. The strong enhancement of the evanescent field in the plasmonic metasurface at the resonant wavelength allows for greater detection sensitivity compared to traditional molecular vibrational spectroscopy. Dielectric metasurfaces are also promising alternatives to enhance the sensitivity of biomedical spectroscopy due to their lower absorption losses, richer resonant modes, and compatibility with CMOS technology. With advancements in nanofabrication technology, an increasing number of materials can serve as substrates for metasurface-enhanced spectroscopy. By adjusting the structural parameters of metasurfaces composed of various materials and structures, the spectrum, wavefront, and polarization of electromagnetic waves can be easily manipulated to meet specific biological detection needs, facilitating colorimetric, terahertz, and chiral sensing, as discussed in Section 5. Overall, metasurface-enhanced spectroscopy offers improved performance and enhanced functionality compared to traditional biomedical detection methods, which is highly significant for biomedical research and disease diagnosis.

Furthermore, based on a comprehensive analysis and synthesis of the most recent literature, we firmly assert that the utilization of metasurfaces will propel biological detection into an unprecedented realm of advancement. Efficient approaches for protein design and structure prediction have garnered significant attention from the scientific community, culminating in the prestigious 2024 Nobel Prize in Chemistry due to their unparalleled potential for enhancing drug screening through targeted protein modulation. Recently, infrared spectroscopy has emerged as a powerful tool for validating cellular-level interactions between drugs and proteins. However, the water environment in cell culture mediums significantly impacts infrared spectroscopy detection. Metasurfaces not only enhance the infrared signal of cell membrane proteins near the substrate but also create a low-water-content space between the cell and metasurfaces, facilitating drug screening through infrared spectroscopy [171], [172], [173], [174], [175]. Meanwhile, recent studies have demonstrated that novel disease markers, such as exosomes and extracellular vesicles, are valuable for the early screening of cancer, as well as cardiovascular and cerebrovascular diseases. However, due to the complexity of their origins, the development of single-vesicle detection technologies is crucial for accurately detecting the expression of specific proteins on the surface of exosomes or extracellular vesicles [176]. Metasurface substrates, with their advantages of strong fluorescence signal amplification and ease of surface chemical modification, are regarded as the most promising approach for detecting these emerging disease markers [177], [178]. Additionally, metasurfaces can enhance the sensitivity and accuracy of biomolecule detection through manipulation and enhancement of the light field, thereby improving the reliability of detection results. Furthermore, they enable simultaneous detection of multiple targets, further enhancing the capability of detection. By integrating metasurfaces with wearable devices, the real-time monitoring of diverse physiological indicators in the human body has been successfully implemented [179], [180], [181], [182], [183]. This integration not only enhances the convenience of biological detection but also offers robust support for personalized medicine and disease prediction, thereby showcasing a remarkable innovation in the realm of biosensing.

While metasurfaces are often highlighted as ideal candidates for enhancing the sensitivity of biomedical spectroscopy, several current challenges that limit their biosensing applications require attention. Their intricate structures can be easily influenced by external environments, and the sample solution and biomolecules involved in biological detection may contaminate or corrode the metasurface, impacting its stability and durability. Additionally, the biocompatibility and safety of metasurface materials are critical considerations; it is essential to ensure that these materials do not release harmful substances during the biological detection process. More importantly, specificity is a crucial aspect of biosensing. If the detection method based on metasurface cannot accurately distinguish the target biomolecule from other similar molecules, it may lead to false detection or missed detection [184], [185], [186]. To address these challenges, it is necessary to explore new materials, processes, and technologies to facilitate the broader applications and further development of metasurfaces in biological detection.

In the future, the application of metasurface in biomedical detection will continue to evolve. As new materials and technologies emerge, the performance of metasurfaces will improve, enabling high-precision detection of a broader range of biomolecules. For example, the combination of metasurfaces and microfluidics has significant potential applications in the field of biological detection. This combination not only leverages the high sensitivity and electromagnetic wave manipulation capabilities of metasurfaces but also benefits from the efficiency and accuracy of microfluidic technology in sample processing, cell manipulation, and detection. Potential applications of this integration in biological detection include (1) enhancing the sensitivity and specificity of detection; (2) enabling real-time, rapid, and portable biological detection; and (3) facilitating multi-marker detection and disease diagnosis [136], [151], [187], [188]. Furthermore, the integration of metasurfaces with artificial intelligence will broaden their application, supporting more efficient biosensing and imaging.
